# Large Vertical Piezoelectricity in a Janus Cr_2_I_3_F_3_ Monolayer

**DOI:** 10.3390/ma15134418

**Published:** 2022-06-22

**Authors:** Haibo Niu, Yachao Liu, Jing Shi, Vei Wang

**Affiliations:** 1 Department of Physics, Xi’an Jiaotong University City College, Xi’an 710018, China; n_haibo@163.com; 2 Department of Applied Physics, School of Science, Xi’an University of Technology, Xi’an 710054, China; liuyachao@xaut.edu.cn (Y.L.); wangvei@xaut.edu.cn (V.W.)

**Keywords:** chromium triiodide, piezoelectric properties, 2D Janus monolayer, vertical polarization, first-principles calculations

## Abstract

Two-dimensional (2D) materials have potential applications in nanoscale sensors and spintronic devices. Herein, motivated by experimental synthesis of a CrI${}_{3}$ monolayer possessing intrinsic magnetism and a Janus MoSSe monolayer with piezoelectricity, we propose a 2D Janus Cr${}_{2}$I${}_{3}$F${}_{3}$ monolayer as a multifunctional material exhibiting both piezoelectricity and ferromagnetism. Using density functional theory calculations, we systematically investigated the structural stability and the electronic, magnetic, and piezoelectric properties of the Janus Cr${}_{2}$I${}_{3}$F${}_{3}$ monolayer. We predicted that a vertical polarization of up to −0.155 × 10${}^{-10}$ C/m is induced in the Cr${}_{2}$I${}_{3}$F${}_{3}$ monolayer due to the breaking of symmetry. The origination mechanism of polarization was demonstrated in terms of a local dipole moment calculated by maximally localized Wannier functions. Meanwhile, it was found that a remarkable piezoelectric response can be produced under a uniaxial strain in the basal plane. The calculated piezoelectric coefficients of the Cr${}_{2}$I${}_{3}$F${}_{3}$ monolayer compare favorably with those of the frequently used bulk piezoelectric materials such as α–quartz and wurtzite AlN. Particularly, the *e*${}_{31}$ and *d*${}_{31}$ values of the Cr${}_{2}$I${}_{3}$F${}_{3}$ monolayer are nearly 10 times as large as that of Mo-based transition metal dichalcogenides. We also found that the magnitude of *e*${}_{31}$ mainly arises from the ionic contribution, while the electronic contribution can be nearly neglected. The considerable piezoelectric response combined with the intrinsic magnetism make the Janus Cr${}_{2}$I${}_{3}$F${}_{3}$ monolayer a potential candidate for novel multifunctional devices integrating both piezoelectric and spintronic applications.

## 1. Introduction

In recent years, low-dimensional functional materials, such as 2D ferromagnetic [1], 2D multiferroic [2], and 2D piezoelectric materials [3], have attracted great attention owing to their fascinating physical characteristics and their potential application in future nano-technologies. As a good example, the chromium triiodide (CrI${}_{3}$) monolayer is the thinnest intrinsic ferromagnetic material that has been successfully synthesized by the mechanical exfoliation method [4]. Most of the studies about 2D CrI${}_{3}$ materials have been focused on their ferromagnetic properties, contributing to the opportunity for the development of nano-spintronic devices [5,6,7]. What is even more enticing is that, if a CrI${}_{3}$ monolayer combines magnetism and piezoelectricity, it will offer considerable potential for integrating microelectronics and spintronics. However, due to the honeycomb structure where chromium atoms are sandwiched between two atomic planes of iodine, a pristine CrI${}_{3}$ monolayer is a non-polar ferromagnetism semiconductor.

Fortunately, although centrosymmetric 2D materials possess no intrinsic piezoelectricity, approaches such as the introduction of defects [8], applying external stress [9] and an electric field [10], surface adsorption [11], and atom intercalation [12], which break centrosymmetry, have been utilized to generate piezoelectric responses in these structures. Besides these approaches, the construction of a Janus structure is a new method that breaks the mirror symmetry. Specifically, the Janus monolayer has the general chemical formula XMY. Because of the different atomic radius and electronegativity of X and Y elements, charge distributions of the M−X layer are not equal to those of the M−Y layer, resulting in obvious spontaneous polarization [13]. Thus, the Janus method paves a way to explore piezoelectric responses in 2D materials [14], and some related research has been implemented. Dong et al. performed a theoretical study and demonstrated strong piezoelectric effects in monolayer and multilayer Janus MXY (M = Mo, W; X/Y = S, Se, and Te) transition metal dichacolgenides (TMDs) [15]. Additionally, such Janus MXY monolayers such as MoSSe have been successfully synthesized in labs, suggesting a possibility for future applications [16].

Consequently, inspired by the Janus monolayer, in which electric polarization can be induced owing to the asymmetry in the atomic and electronic structure, in this work, a Cr${}_{2}$I${}_{3}$F${}_{3}$ monolayer is designed by replacing iodine atoms at the top layer in the CrI${}_{3}$ monolayer with fluorine atoms, which have the largest electronegativity and exhibit the strongest attractive electron ability among all elements [17]. As a result, the significant inversion symmetry breaking of this configuration will induce an evident out-of-plane spontaneous polarization. Moreover, because the intrinsic difference in the electronegativity of atoms in the unit cell defines the magnitude of the piezoelectric response [18], a considerable piezoelectric response will be generated in the Janus Cr${}_{2}$I${}_{3}$F${}_{3}$ monolayer. Therefore, the Cr${}_{2}$I${}_{3}$F${}_{3}$ monolayer becomes a 2D multi-functional material with both piezoelectric and ferromagnetic properties [19]. The diversity of the novel characteristics imparted to the Cr${}_{2}$I${}_{3}$F${}_{3}$ monolayer would significantly expand application fields such as catalysis [20], spintronics [21], energy harvesting [18], and sensing [22].

In this work, the structural, electronic, magnetic, and piezoelectric properties of the Janus Cr${}_{2}$I${}_{3}$F${}_{3}$ monolayer are investigated by using first-principles calculations. It is found that the magnetic properties of the Janus Cr${}_{2}$I${}_{3}$F${}_{3}$ monolayer is almost the same as those of the CrI${}_{3}$ monolayer, revealing the preservation of magnetism. On the other hand, a large electric polarization is induced along the out-of-plane direction. The subsequent calculations indicate that the Janus Cr${}_{2}$I${}_{3}$F${}_{3}$ monolayer shows an excellent piezoelectric response in the vertical direction, which is distinctly better than that of many TMDs (e.g., MoSSe and MoSeTe), and is as good as that of the frequently used 3D piezoelectric materials such as α–quartz and wurtzite AlN. Furthermore, the origination mechanism of polarization and the strain coefficient were explored in terms of the local dipole moment computed by maximally localized Wannier functions (MLWFs). Finally, to further enhance the piezoelectric response, a study of the piezoelectric coefficients as a function of biaxial strain (ε) varying from −4.0% to 4.0% was performed.

## 2. Materials and Methods

### 2.1. Computation Details

Our total energy and electronic structure calculations were performed employing the Vienna ab initio simulation package (VASP) [23]. The electron–ion interaction was described by the projected augmented wave (PAW) method [24]. The exchange and correlation were treated with generalized gradient approximation in the Perdew–Burke–Ernzerhof (PBE) form [25]. Because the band gaps of semiconductors are often underestimated by DFT calculations with local or semi-local exchange-correlation functionals, some of the electronic structure calculations were also performed using the Heyd–Scuseria–Ernzerhof (HSE06) hybrid functional [26,27]. A cutoff energy of 600 eV was adopted for the plane wave basis set, yielding a total energy convergence better than 1 meV/atom. A Γ-centered 9 × 9 × 1 *k*-point mesh is used for the unit cell optimization. Structures are fully relaxed until the force acting on each atom is reduced to less than 0.001 eV/Å. The vacuum thickness between two neighbor slabs is 15 Å in the *z* direction to avoid mirror interactions. The VASPKIT code is used to post-process the calculated data obtained by VASP code [28]. The electric polarization of the systems are computed by using Wannier90 code [29]. The phonon dispersions were performed utilizing a finite difference approach, as implemented in the Phonopy package, in which a 4 × 4 × 1 supercell is employed [30,31]. The Curie temperature (*T*${}_{C}$) is evaluated by using the Metropolis Monte Carlo (MC) simulations implemented in mcsolver code [32,33].

### 2.2. Electric Polarization and Piezoelectricity

Based on the modern polarization theory, electric polarization *P* of a periodic 2D structure can be calculated by the MLWF method, expressed as [29,34]

(1) $$P=P_{\mathrm{ion}}+P_{\mathrm{ele}}=\frac{e}{S}\left[\sum_{i}q_{i}r_{i}+\sum_{j}q_{j}w_{j}\right],$$

where *P*${}_{\mathrm{ion}}$ and *P*${}_{\mathrm{ele}}$ represent the ionic and electronic contributions, respectively. Correspondingly, $q_{i}$ and $r_{i}$ represent the charge quantity and position of the *i*th ion, and $q_{j}$ and $w_{j}$ denote the charge quantity and position of the *j*th Wannier function center (WFC), respectively. Since *S* is the area of the whole structure, *P* obtained by Equation (1) stands for the average polarization of the whole system. Nevertheless, it is worth noting that the MLWF method also demonstrates an insightful local description of the polarization [35,36]. Specifically, because the MLWF method partitions the system into localized WFCs and ionic charges, one can define the dipole moment of the local region of the structure, providing a local description of polarization, such as the layer-by-layer polarization analysis. Thus, the MLWF method is of particular useful for discussing not only the polarization variation within the same system, but also the polarization difference between the structures in different dimensions (e.g., the 2D phase and the bulk phase), exhibiting significant advantages in the study of polarization in 2D materials.

The piezoelectric effect can be described by third-rank tensors *e*${}_{ijk}$ and *d*${}_{ijk}$ as

(2) $$\begin{array}{c} e_{ijk}=\partial P_{i}/\partial \varepsilon_{jk}\\ d_{ijk}=\partial P_{i}/\partial \sigma_{jk}, \end{array}$$

where *P*${}_{i}$ is the polarization of the structure, and $\varepsilon_{jk}$ and $\sigma_{jk}$ are strain and stress, respectively. Since the Janus Cr${}_{2}$I${}_{3}$F${}_{3}$ monolayer possesses C${}_{3v}$ symmetry, and the stress and strain can only be applied within the basal plane, the piezoelectric tensors can be written as [15]

$$e_{ik}=\left(\begin{array}{ccc} e_{11} & -e_{11} & 0\\ 0 & 0 & -0.5e_{11}\\ e_{31} & e_{31} & 0 \end{array}\right)$$

$$d_{ij}=\left(\begin{array}{ccc} d_{11} & -d_{11} & 0\\ 0 & 0 & -d_{11}\\ d_{31} & d_{31} & 0 \end{array}\right)$$

The *e*${}_{ik}$ and *d*${}_{ij}$ are related by the elastic stiffness tensor *C*${}_{jk}$:

(3) $$e_{ik}=C_{jk}d_{ij}.$$

In our calculations, the values of *e*${}_{11}$ and *e*${}_{31}$ are evaluated by a linear fitting of the polarization change along the *x* and *z* directions with respect to the strain $\varepsilon_{1}$. Afterward, the corresponding values of *d*${}_{11}$ and *d*${}_{31}$ are computed by

(4) $$\begin{array}{c} d_{11}=e_{11}/\left(C_{11}-C_{12}\right)\\ d_{31}=e_{31}/\left(C_{11}+C_{12}\right). \end{array}$$

## 3. Results and Discussion

### 3.1. Structural Stability

**Figure 1:**
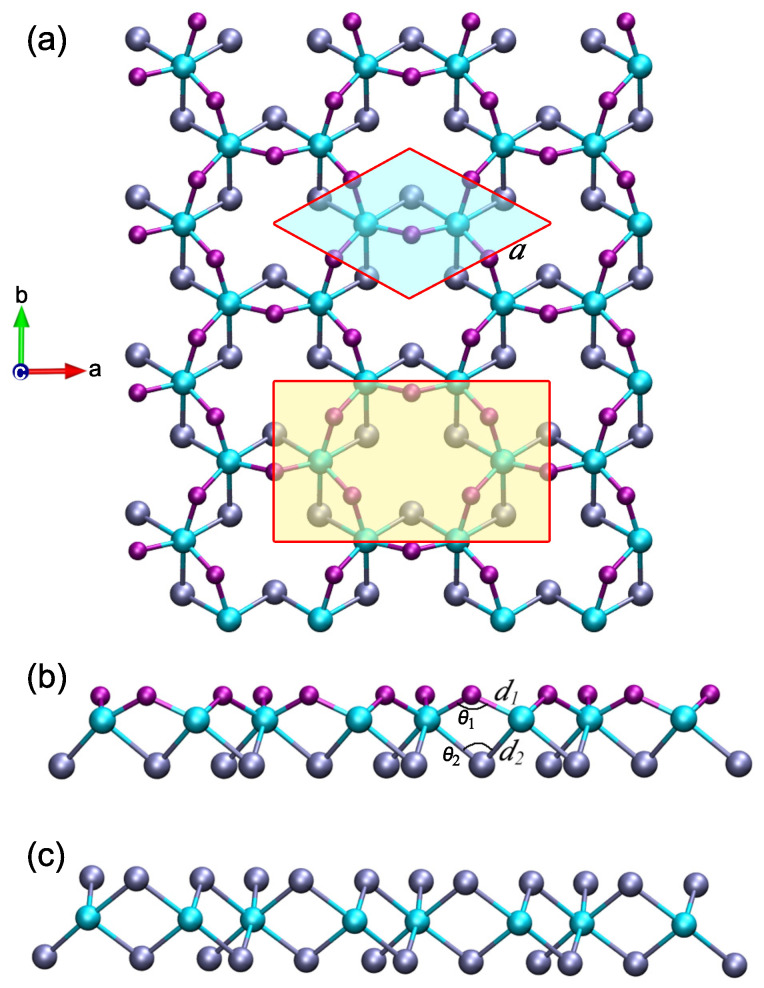
Atomic structures of (**a**) the top view and (**b**) the side view of the relaxed Janus Cr${}_{2}$I${}_{3}$F${}_{3}$ monolayer. The hexagonal primitive cell is highlighted in light cyan. The orthorhombic unit cell used in the piezoelectric coefficient calculation is marked in light yellow. (**c**) Side view of the relaxed CrI${}_{3}$ monolayer. The chromium, iodine, and fluorine atoms are displayed by the cyan, ice-blue, and purple balls, respectively.

As evidence of the reliability of our simulation, we start by optimizing the unit cell of the CrI${}_{3}$ monolayer. Our calculated results show that the equilibrium lattice constant and the Cr–I–Cr bond angle are 7.002 Å and 95.25${}^{\circ }$, which agrees well with previous reports [37,38,39,40,41]. The Janus Cr${}_{2}$I${}_{3}$F${}_{3}$ monolayer is then constructed by replacing iodine atoms in the top layer with fluorine atoms, consisting of three atomic layers of F–Cr–I from top to bottom. The atomic structure of the Cr${}_{2}$I${}_{3}$F${}_{3}$ monolayer is presented in Figure 1, which shows that the fluorine atoms apparently move toward the chromium layer owing to the largest electronegativity. Consequently, the top F–Cr bond (*d${}_{1}$* = 2.057 Å) is notably smaller than the bottom I–Cr bond (*d${}_{2}$* = 2.695 Å), and the top Cr–F–Cr bond angle ($\theta_{1}$ = 122.48${}^{\circ }$) is significantly larger than the bottom Cr–I–Cr bond angle ($\theta_{2}$ = 84.00${}^{\circ }$) listed in Table 1, revealing an evident asymmetry in the atomic structure. Interestingly, although the same kind of parameters, it is found that the I–Cr bond and the Cr–I–Cr bond angle of the Janus Cr${}_{2}$I${}_{3}$F${}_{3}$ monolayer are different from those of the CrI${}_{3}$ monolayer (2.695 Å versus 2.736 Å, 84.00${}^{\circ }$ versus 95.25${}^{\circ }$). Therefore, the bottom iodine atoms also have a slight movement toward the chromium layer under the influence of the top fluorine atoms. As a consequence, the calculated lattice parameter of the Cr${}_{2}$I${}_{3}$F${}_{3}$ monolayer (*a* = 6.246 Å) is significantly smaller than that of the CrI${}_{3}$ monolayer. In fact, the lattice parameter of the Cr${}_{2}$I${}_{3}$F${}_{3}$ monolayer lies between that of the CrI${}_{3}$ monolayer and the CrF${}_{3}$ monolayer.

**Figure 2:**
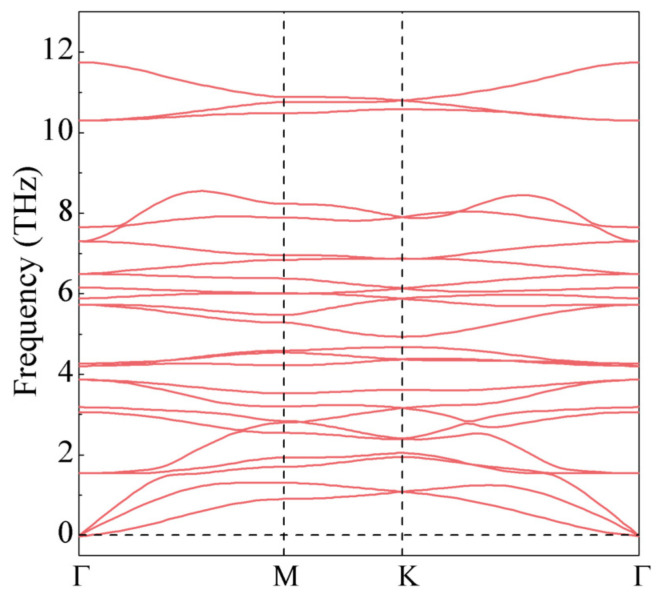
Phonon dispersion of the Janus Cr${}_{2}$I${}_{3}$F${}_{3}$ monolayer.

We next studied the stability of the Cr${}_{2}$I${}_{3}$F${}_{3}$ monolayer by employing phonon dispersion calculations. As displayed in Figure 2, no imaginary vibrational frequency exists in the first Brillouin zone, validating the dynamical stability of the structure. The thermodynamic stability is further verified by calculating the formation energy (ΔH), which can be written as

(5) $$\Delta H=\left(E_{\mathrm{Janus}}-n_{\mathrm{Cr}}\times E_{\mathrm{Cr}}-n_{I}\times E_{I}-n_{F}\times E_{F}\right)/\left(n_{\mathrm{Cr}}+n_{I}+n_{F}\right),$$

where $E_{\mathrm{Janus}}$ is the total energy of the Janus Cr${}_{2}$I${}_{3}$F${}_{3}$ monolayer; $E_{\mathrm{Cr}}$, $E_{I}$, and $E_{F}$ are the energies per atom of the chromium, iodine, and fluorine bulk phases, respectively; $n_{\mathrm{Cr}}$, $n_{I}$, and $n_{F}$ represent the atom numbers of chromium, iodine, and fluorine, respectively. The computed formation energy has a negative value of −1.31 eV, confirming that the Janus Cr${}_{2}$I${}_{3}$F${}_{3}$ monolayer is energetically favorable.

**Figure 3:**
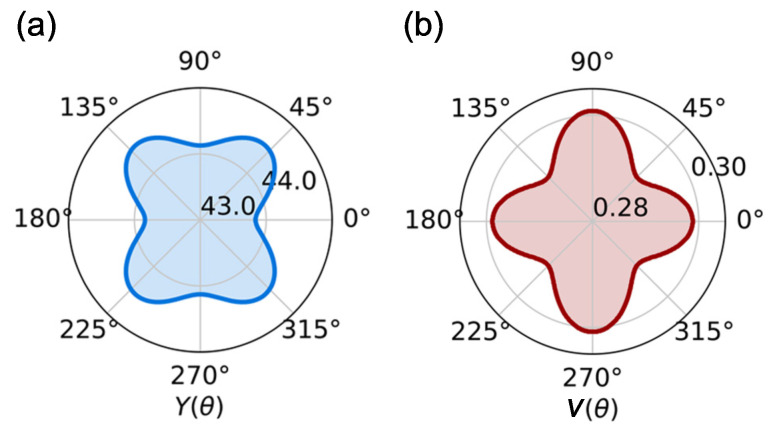
(**a**) The Young’s modulus and (**b**) Poisson’s ratio of the Janus Cr${}_{2}$I${}_{3}$F${}_{3}$ monolayer as a function of the angle θ.

For the Janus Cr${}_{2}$I${}_{3}$F${}_{3}$ monolayer with a hexagonal symmetry, there are two independent elastic constants *C*${}_{11}$ and *C*${}_{12}$. Table 2 shows that the calculated *C*${}_{11}$ (48.762 N/m) and *C*${}_{12}$ (14.668 N/m) are both positive. The result fulfills the Born stability criteria, namely, $C_{11}C_{22}-C_{12}^{2}>0$, illustrating that the Janus Cr${}_{2}$I${}_{3}$F${}_{3}$ monolayer has good mechanical stability. Note that the elastic constants of the Janus Cr${}_{2}$I${}_{3}$F${}_{3}$ monolayer are evidently smaller than those of the monolayers listed in Table 2 except for the CrI${}_{3}$ monolayer. Therefore, the Janus Cr${}_{2}$I${}_{3}$F${}_{3}$ monolayer has excellent flexibility, which is much desired to improve micro flexible piezoelectric devices. Moreover, such excellent flexibility provides an attractive possibility to tune physical properties such as piezoelectricity through strain engineering. Based on the above-calculated elastic constants, Young’s modulus Y(θ) and Poisson’s ratio ν(θ) as a function of in-plane θ can be expressed as [42]

(6) $$\begin{array}{c} Y\left(\theta \right)=\frac{C_{11}C_{22}-C_{12}^{2}}{C_{22}\cos^{4}\theta +A\cos^{2}\theta \sin^{2}\theta +C_{11}\sin^{4}\theta },\\ \nu \left(\theta \right)=\frac{C_{12}cos^{4}\theta -B\cos^{2}\theta \sin^{2}\theta +C_{12}\sin^{4}\theta }{C_{22}\cos^{4}\theta +A\cos^{2}\theta \sin^{2}\theta +C_{11}\sin^{4}\theta }, \end{array}$$

where $A=\left(C_{11}C_{22}-C_{12}^{2}\right)/C_{66}-2C_{12}$ and $B=C_{11}+C_{22}-\left(C_{11}C_{22}-C_{12}^{2}\right)/C_{66}$. As described in Figure 3, it can be seen that both Young’s modulus Y(θ) and Poisson’s ratio ν(θ) have a weak anisotropy character due to the absence of mirror symmetry in the Janus monolayer. Specifically, Y(θ) ranges from 43.838 to 44.498 N/m, and ν(θ) varies from 0.292 to 0.301.

### 3.2. Electronic and Magnetic Properties

**Figure 4:**
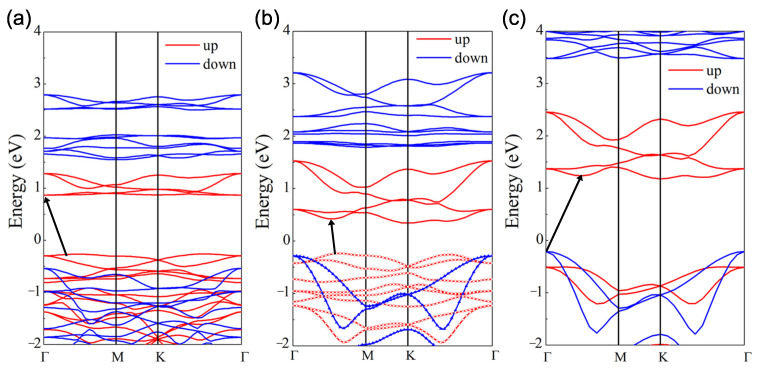
GGA energy band structure of the (**a**) CrI${}_{3}$ and (**b**) Cr${}_{2}$I${}_{3}$F${}_{3}$ monolayers. (**c**) HSE energy band structure of the Cr${}_{2}$I${}_{3}$F${}_{3}$ monolayer. The red and blue lines represent the spin-up and spin-down channels. The Fermi level is set as 0 eV. The energy band gap between VBM and CBM is indicated by the black arrow. The Wannier interpolation valence band (plotted by circle and triangle) is included in (**b**) for comparison.

Figure 4b shows the PBE-calculated band structure of the Janus Cr${}_{2}$I${}_{3}$F${}_{3}$ monolayer, of which a non-zero band gap of 0.58 eV indicates that it is still a semiconductor. Specifically, both the conduction band minimum (CBM) and the valence band maximum (VBM) come from spin-up channels and locate at different *k* points on the Γ-M path, leading to an indirect band gap. Comparing this with the CrI${}_{3}$ monolayer with a band gap of 1.13 eV displayed in Figure 4a, we see an evident reduction in band gap due to a downward shift of CBM for the Janus Cr${}_{2}$I${}_{3}$F${}_{3}$ monolayer. On the other hand, since the PBE-functional calculation can remarkably underestimate the band gap, we also calculated the band structure utilizing the HSE06 functional, and the result is plotted in Figure 4c. It can be seen that the band structure calculated by HSE06 presents similar characteristics to the band structure obtained by the PBE method, as shown in Figure 4b,c. The band gap calculated by HSE06 is expanded to 1.39 eV, along with the upward shifting of the conduction bands. It is also worth noting that the spin-down states contribute to the VBM in the HSE06 results, while the spin-up states contribute to both the CBM and VBM in the PBE calculations.

**Figure 5:**
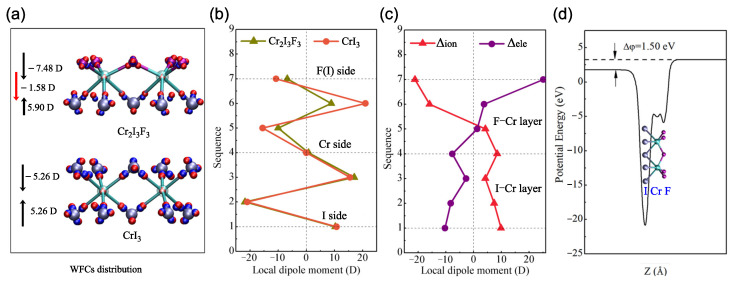
(**a**) WFC distribution of the Cr${}_{2}$I${}_{3}$F${}_{3}$ and CrI${}_{3}$ monolayers. The small red and blue balls represent the WFCs Wanniered from the spin-up and spin-down channels, respectively. (**b**) Local dipole moment analysis in sequence from the fluorine side to the iodine side along the *z* direction. (**c**) The electronic ($\Delta P_{\mathrm{ele}}$) and ionic ($\Delta P_{\mathrm{ion}}$) contributions to the polarization of the Cr${}_{2}$I${}_{3}$F${}_{3}$ monolayer. (**d**) The planar average of the electrostatic potential energy of the Cr${}_{2}$I${}_{3}$F${}_{3}$ monolayer.

Because the inversion symmetry is broken in the Cr${}_{2}$I${}_{3}$F${}_{3}$ monolayer, the charge distribution is different from that of the CrI${}_{3}$ monolayer. We further investigated the charge rearrangement through the distribution of WFCs. To calculate the WFCs accurately, we considered all of the occupied valence bands as a composite group to construct the MLWFs for spin-up and spin-down channels, respectively. After that, a comparison between the Wannier interpolation band and the traditional DFT results was made to assess the computed MLWFs. It can be seen from Figure 4b that the two band structures are virtually indistinguishable, revealing a good Wannierization. Figure 5a exhibits the WFC distribution. We can see that each fluorine and iodine atom are closely surrounded by four spin-up and spin-down WFCs Wanniered from *s* and *p* bands of the atoms. This means that electrons transform from chromium atoms to fluorine and iodine atoms, evidencing the strong ionicity of the I–Cr bond and the F–Cr bond. Nevertheless, it is found that WFCs are much closer to fluorine than to iodine atoms, implying the stronger ionicity of the F–Cr bond and the difference in WFC distribution for the F–Cr layer and the I–Cr layer. Besides the WFCs nearby the fluorine and iodine atoms, the remaining spin-up WFCs are centered at the chromium atoms because they are Wanniered from the Cr 3*d* bands. It is due to the strong localization of the Cr *d*-orbitals that these WFCs have the same location as the chromium atoms. Therefore, the WFC distribution intuitively illustrates the electronic structure asymmetry of the Cr${}_{2}$I${}_{3}$F${}_{3}$ monolayer.

The two faces of the Cr${}_{2}$I${}_{3}$F${}_{3}$ monolayer are composed by fluorine and iodine atoms, respectively, which directly break the inversion symmetry and the out-of-plane mirror symmetry of the structure [43]. Due to the presence of structural asymmetry in the Cr${}_{2}$I${}_{3}$F${}_{3}$ monolayer, the difference in the electronegativity of the fluorine and iodine atoms cause the formation of a dipole across the plane (as shown in Figure 5d), so a vertical polarization is induced in the Cr${}_{2}$I${}_{3}$F${}_{3}$ monolayer. According to the WFC distribution, we carried out a local dipole analysis for the F–Cr layer and the I–Cr layer in the primitive cell (8 atoms) along the *z* direction. Note that each WFC contains one electron due to spin degeneracy, namely, $q_{i}$ = −1 in Equation (1). From Figure 5a, one can see that the dipole moment of the F–Cr layer and the I–Cr layer is −7.48 D and 5.90 D, respectively. Consequently, a net vertical dipole moment of −1.58 D pointing along the direction from fluorine to iodine is induced. This value corresponds to an out-of-plane polarization of *P* = −0.155 × 10${}^{-10}$ C/m, which is two times larger than that of the usual Janus TMD monolayers reported in a previous study [45]. To obtain deep insight into the origination of polarization, we compute the local dipole moment in sequence along the *z* direction from the fluorine side to the iodine side. Compared with those of the CrI${}_{3}$ monolayer displayed in Figure 5b, it can be seen that the dipole moments in the I–Cr layer are nearly identical, whereas a noticeable difference arises in the F–Cr layer, demonstrating that the F–Cr layer mainly accounts for the polarization of the Janus configuration. Furthermore, we calculated the electronic ($\Delta P_{\mathrm{ele}}$) and ionic ($\Delta P_{\mathrm{ion}}$) contribution to the polarization using the CrI${}_{3}$ monolayer as the reference structure. As displayed in Figure 5c, $\Delta P_{\mathrm{ion}}$ (−2.82 D) is two times larger than $\Delta P_{\mathrm{el}}$ (1.25 D). Accordingly, the atomic relaxation of the fluorine atoms plays a critical role in the polarization formation of the Cr${}_{2}$I${}_{3}$F${}_{3}$ monolayer. Additionally, the origination of the net dipole moment can also be understood by the planar average of the electrostatic potential energy represented in Figure 5d. Due to the charge redistribution in the Janus monolayer structure, the fluorine side has a potential energy about 1.50 eV higher than that of the iodine side, resulting in an electric field from fluorine to iodine that coincides with the result obtained by the above MLWF method.

The magnetic properties of the Cr${}_{2}$I${}_{3}$F${}_{3}$ monolayer were also explored by means of WFCs. Firstly, the magnetic moment of the Cr${}_{2}$I${}_{3}$F${}_{3}$ monolayer was inspected by WFC distribution. In particular, observing the WFCs nearby fluorine and iodine atoms (see Figure 5a), we can see that the numbers and locations of the spin-down WFCs are the same as those of the spin-up WFCs, which leads to a magnetic moment counterbalance. Thus, the magnetic moment of the Cr${}_{2}$I${}_{3}$F${}_{3}$ monolayer mainly arises from the three spin-up WFCs located at the chromium position, giving rise to 3.0 $\mu_{B}$ per chromium atom. This value agrees well with that of the CrI${}_{3}$ monolayer computed by DFT theory, indicating that the Cr${}_{2}$I${}_{3}$F${}_{3}$ monolayer preserves the intrinsic magnetism [7,46]. Secondly, by employing the PBE functional combined with spin-orbit coupling (SOC), we calculated the magnetic anisotropy energy (MAE) by the energy difference between the out-of-plane and in-plane magnetization directions of the Cr${}_{2}$I${}_{3}$F${}_{3}$ monolayer (expressed as *E*${}_{z}$-*E*${}_{x}$), and we list the result in Table 3. The obtained negative result of the CrI${}_{3}$ and CrF${}_{3}$ monolayer exhibits the out-of-plane magnetic anisotropy. However, the positive result of the Cr${}_{2}$I${}_{3}$F${}_{3}$ monolayer shows an in-plane magnetic anisotropy. To confirm the easy axis of the Cr${}_{2}$I${}_{3}$F${}_{3}$ monolayer, we also computed the MAE utilizing the HSE06 functional and obtained a result of 2.171 meV per chromium atom. Thus, the easy axis of the Cr${}_{2}$I${}_{3}$F${}_{3}$ monolayer lies in the xy-plane, which is consistent with our PBE calculations. Moreover, this considerable MAE makes the Cr${}_{2}$I${}_{3}$F${}_{3}$ monolayer a promising candidate for low-dimensional magneto-electronic applications.

**Figure 6:**
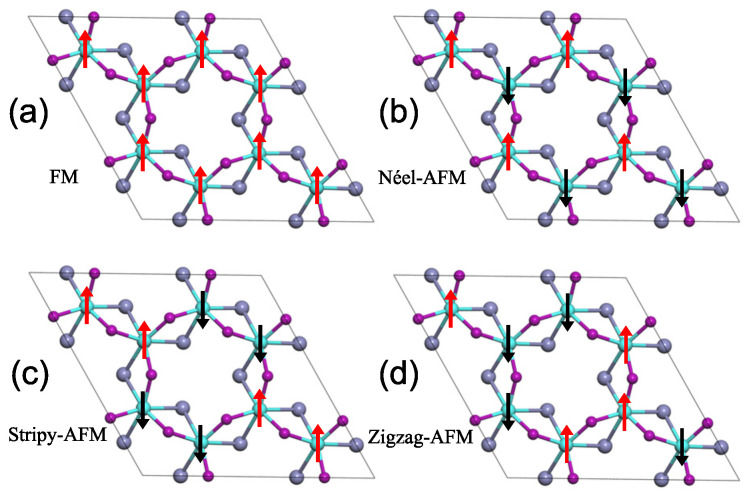
Four magnetic orders of the Cr${}_{2}$I${}_{3}$F${}_{3}$ monolayer (2 × 2 × 1 supercell). (**a**) FM, (**b**) N$\overset{´}{e}$el-AFM, (**c**) Stripy-AFM, and (**d**) Zigzag-AFM. The red (black) arrows represent the spin-up (spin-down).

**Figure 7:**
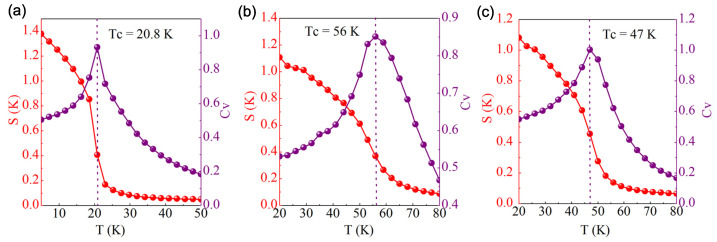
Simulated spin structure factor S(k) and specific heat C${}_{V}$ as a function of temperature from Monte Carlo simulations. (**a**) CrF${}_{3}$, (**b**) CrI${}_{3}$, and (**c**) Cr${}_{2}$I${}_{3}$F${}_{3}$ monolayer.

The Curie temperature (*T*${}_{C}$) of the Cr${}_{2}$I${}_{3}$F${}_{3}$ monolayer was evaluated by using the Metropolis Monte Carlo (MC) simulations implemented in mcsolver code [32,33]. To perform the evaluation, the exchange coupling *J*${}_{\mathrm{ex}}$, consisting of the nearest neighbor (NN) exchange interaction *J*${}_{1}$, the next nearest neighbor (NNN) interaction *J*${}_{2}$, and the third nearest neighbour (3NN) interaction *J*${}_{3}$, is firstly calculated by combining DFT calculations with the Heisenberg spin Hamiltonian [7]. Specifically, we considered the FM order and three possible AFM configurations, namely, N$\overset{´}{e}$el-AFM, Stripy-AFM, and Zigzag-AFM, which are depicted in Figure 6. After that, by comparing the magnetic energy of these four configurations, *J*${}_{\mathrm{ex}}$ could be acquired [5,7], which is listed in Table 3. As shown in Figure 7, our calculated *T*${}_{C}$ of the Cr${}_{2}$I${}_{3}$F${}_{3}$ monolayer is about 47 K, which is slightly smaller than that of the CrI${}_{3}$ monolayer but evidently larger than that of the CrF${}_{3}$ monolayer. Moreover, using the energy of the FM state as the reference, the energy differences are 37.8, 59.4, and 61.1 meV for the N$\overset{´}{e}$el-AFM, Stripy-AFM, and Zigzag-AFM states, respectively. Thus, the most stable magnetic state is the FM state. This also demonstrates that ferromagnetism in the CrI${}_{3}$ monolayer is retained by using fluorine element substitution to construct the Janus Cr${}_{2}$I${}_{3}$F${}_{3}$ monolayer.

### 3.3. Piezoelectricity Properties

**Figure 8:**
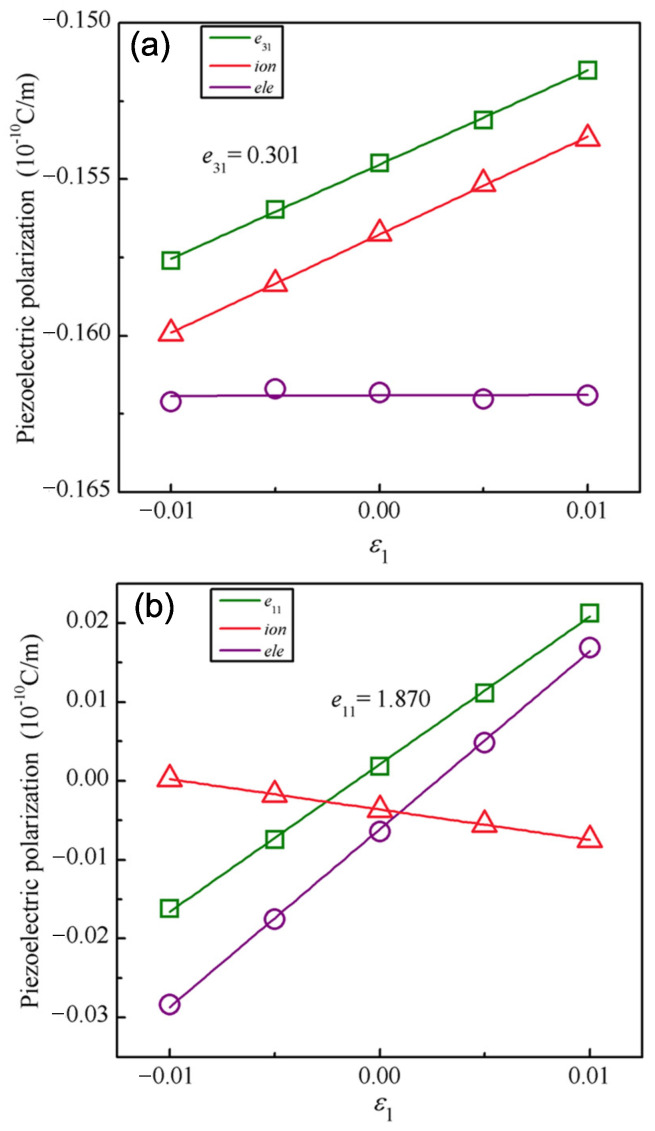
Piezoelectric stress coefficients (**a**) *e*${}_{31}$ and (**b**) *e*${}_{11}$ obtained by linear changes in out-of-plane and in-plane piezoelectric polarizations under a uniaxial strain $\varepsilon_{1}$. The ionic and electronic contribution to *e*${}_{31}$ and *e*${}_{11}$ is also listed for comparison. To conveniently compare the three, the ionic and electronic contribution is properly rescaled.

Now we turn to explore the piezoelectricity of the Janus Cr${}_{2}$I${}_{3}$F${}_{3}$ monolayer. As displayed in Figure 8, the piezoelectric stress coefficient *e*${}_{31}$ and *e*${}_{11}$ are calculated by a linear fitting of the piezoelectric polarization as a function of a uniaxial in-plane strain $\varepsilon_{1}$, which is changed from –1.0% to 1.0% with a step size of 0.5%. In our study, the atomic and electronic structure are both fully relaxed at each stress level to acquire the equilibrium state. Afterwards, the electric polarization of each state is evaluated by using the MLWF method. The slopes derived from a least-square linear fitting of the piezoelectric polarization at the equilibrium state correspond to the piezoelectric stress coefficients. In terms of the elastic stiffness coefficients of *C*${}_{11}$ and *C*${}_{12}$, the piezoelectric strain coefficients *d*${}_{31}$ and *d*${}_{11}$ are computed based on Equation (4).

The piezoelectric stress coefficient *e*${}_{31}$ for different Janus Mo/W-based transition metal dichalcogenides (MoSSe, In${}_{2}$SSe, MoSTe, MoSeTe, WSeTe, WSSe, and WSTe) are provided in Table 2, as a comparison with the Cr${}_{2}$I${}_{3}$F${}_{3}$ monolayer. For transition metal dichalcogenides, the Mo-based transition metal dichalcogenides (MoSSe, MoSTe, and MoSeTe) present the largest piezoelectric coefficients *e*${}_{31}$ (0.032 × 10${}^{-10}$, 0.038 × 10${}^{-10}$, and 0.037 × 10${}^{-10}$ C/m, respectively) and *d*${}_{31}$ (0.020, 0.028, and 0.030 pm/V, respectively). It is found that the Cr${}_{2}$I${}_{3}$F${}_{3}$ monolayer presents significantly larger piezoelectric coefficients *e*${}_{31}$ (0.301 × 10${}^{-10}$ C/m) and *d*${}_{31}$ (0.475 pm/V) than the previous studied transition metal dichalcogenides in Table 2, and the *e*${}_{31}$ and *d*${}_{31}$ values are nearly 10 times as large as that of Mo-based transition metal dichalcogenides. The *e*${}_{11}$ and *d*${}_{11}$ of the Cr${}_{2}$I${}_{3}$F${}_{3}$ monolayer are calculated to be 1.870 × 10${}^{-10}$ C/m and 5.485 pm/V, respectively. The *e*${}_{11}$ value of the Cr${}_{2}$I${}_{3}$F${}_{3}$ monolayer is comparable to the experimental results of the widely studied h-BN (2.91 × 10${}^{-10}$ C/m) [47] and MoS${}_{2}$ (2.9 × 10${}^{-10}$ C/m) monolayers [48], which demonstrates the application potential for piezoelectric devices. Additionally, the *d*${}_{11}$ of the Cr${}_{2}$I${}_{3}$F${}_{3}$ monolayer is already as good as those of frequently used 3D piezoelectric materials, such as α–quartz (*d*${}_{11}$ = 2.27 pm/V) [49] and AlN (*d*${}_{33}$ = 5.4 pm/V) [50]. As a consequence, the Janus Cr${}_{2}$I${}_{3}$F${}_{3}$ monolayer has an outstanding piezoelectric response, providing a potential opportunity for piezoelectric applications based on 2D materials.

The Cr${}_{2}$I${}_{3}$F${}_{3}$ monolayer possesses a remarkable piezoelectric response, which is benefiting from the high intrinsic out-of-plane spontaneous polarization and the strong local distortion in the Cr${}_{2}$I${}_{3}$F${}_{3}$ lattice. The high electronegativity difference between the fluorine and iodine face induces strong out-of-plane spontaneous polarization in the Cr${}_{2}$I${}_{3}$F${}_{3}$ monolayer (−0.155 × 10${}^{-10}$ C/m or −1.58 D), which is significantly larger than surface defected I${}_{V}$-CrI${}_{3}$ (0.13 D) [8]. In the Cr${}_{2}$I${}_{3}$F${}_{3}$ monolayer, the F–Cr and I–Cr bond lengths are 2.057 Å and 2.695 Å, respectively. The corresponding Cr–F–Cr and I–Cr–I bond angles are 122.48${}^{\circ }$ and 84.00${}^{\circ }$, respectively. The significant structural difference between the F–Cr and I–Cr faces indicates that strong intrinsic strain is induced in the Cr${}_{2}$I${}_{3}$F${}_{3}$ lattice, which could promote the ionic displacement and significantly improve the piezoelectric response [51,52,53].

To obtain a deep insight into the mechanism of the piezoelectric response under the uniaxial strain, we further performed an analysis from the ionic and electronic contribution. The ionic and electronic contributions to the coefficients *e*${}_{31}$ were calculated by the linear fitting method, respectively, as shown in Figure 8a. The ionic contribution to the *e*${}_{31}$ is 0.299 × 10${}^{-10}$ C/m, while the electronic contribution is almost zero (0.002 × 10${}^{-10}$ C/m). This shows that the total *e*${}_{31}$ response is dominated by the ionic contribution, while the electronic contribution is negligible. Conversely, for *e*${}_{11}$, the ionic and electronic contributions included in Figure 8b both change significantly and maintain the opposite part under the uniaxial strain $\varepsilon_{1}$ along the in-plane direction. Owing to a larger slope (2.255 × 10${}^{-10}$ C/m) than that (−0.385 × 10${}^{-10}$ C/m) of the ionic contribution, the electronic contribution is mainly responsible for *e*${}_{11}$. Therefore, these findings are of a certain guiding significance for regulating the piezoelectricity along the in-plane and out-of-plane directions, respectively. For instance, the in-plane polarization can be effectively adjusted by an applied external electric field [10].

On the other hand, to enhance the piezoelectric response along the out-of-plane direction, apart from the method by stacking layers to construct multilayers [15,54], a strain varied in a large range can be applied. Meanwhile, the intrinsic monolayer configuration is also reserved. The strain engineering has been shown to be a very effective way to enhance the piezoelectric properties [9,55]. In fact, stain engineering is more preferable for the Janus monolayer Cr${}_{2}$I${}_{3}$F${}_{3}$ because of the inherent smaller elastic constant.

**Figure 9:**
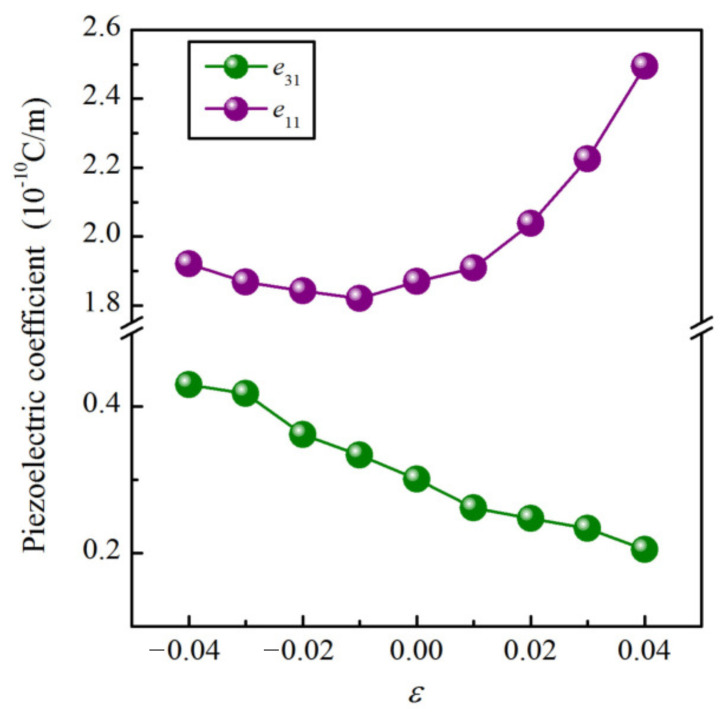
*e*${}_{31}$ and *e*${}_{11}$ as a function of biaxial strain ε.

It is well known that a large out-of-plane piezoelectric response is of particular importance for practical applications. Herein, according to the discussion above, we explored the piezoelectric properties of the Janus Cr${}_{2}$I${}_{3}$F${}_{3}$ monolayer as a function of biaxial strain (ε) varying from −4.0% to 4.0%, which could be easily realized in experiments. Figure 9 displays the obtained *e*${}_{31}$ and *e*${}_{11}$. It can be seen that the largest *e*${}_{31}$ (0.430 × 10${}^{-10}$ C/m) is obtained under an ε of −4.0%, which is increased by 43%. Furthermore, it seems that *e*${}_{31}$ monotonically increases under the compression strain and decreases under the tension strain. Moreover, the largest *e*${}_{11}$ of 2.49 × 10${}^{-10}$ C/m is acquired under an ε of 4.0%, which is increased by 33%.

**Figure 10:**
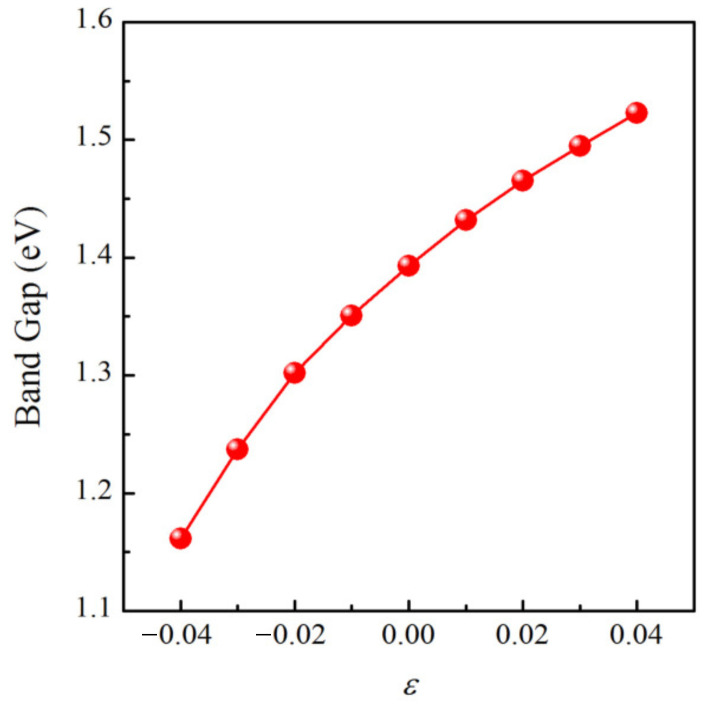
The HSE06 band gap as a function of biaxial strain ε.

Since the piezoelectric material should have a rational band gap to avoid current leakage, we further calculated the band gap as a function of biaxial strain employing HSE06 code [56]. As plotted in Figure 10, a none zero band gap smaller than 1.55 eV can be found under the biaxial strain within 4.0%, indicating that the material is still a semiconductor. Moreover, the band gap monotonically increases when the biaxial strain changes from −4.0% to 4.0%.

## 4. Conclusions

In summary, we have studied the structural, electronic, magnetic, and piezoelectric properties of the Janus Cr${}_{2}$I${}_{3}$F${}_{3}$ monolayer using first-principles calculations accompanied by MLWFs. Based on the phonon, formation energy, and mechanical calculations, we proved that the Janus Cr${}_{2}$I${}_{3}$F${}_{3}$ monolayer is thermally, energetically, and mechanically stable and possesses excellent flexibility. The magnetic properties of the Cr${}_{2}$I${}_{3}$F${}_{3}$ monolayer were studied; the magnetic moment in the FM ground state is 6.0 $\mu_{B}$ per unit cell, and the corresponding *T*${}_{C}$ value was calculated to be 47 K. This shows that the ferromagnetism observed in the CrI${}_{3}$ monolayer is retained in the Cr${}_{2}$I${}_{3}$F${}_{3}$ monolayer. The PBE and HSE06 functional calculations show that the Cr${}_{2}$I${}_{3}$F${}_{3}$ monolayer is a semiconductor with an indirect band gap of 0.58 eV and 1.39 eV, respectively. The charge rearrangement and atomic relaxation mainly occurred in the F–Cr layer, leading to a large vertical polarization of up to −0.155 × 10${}^{-10}$ C/m. Remarkable in-plane and out-of-plane piezoelectric responses were found under a uniaxial strain in the basal plane. The obtained strain coefficient *e*${}_{31}$ was up to 0.301 × 10${}^{-10}$ C/m, which is significantly larger than the previously studied 2D transition metal dichalcogenides MoSSe (0.032 × 10${}^{-10}$ C/m), MoSTe (0.038 × 10${}^{-10}$ C/m), and MoSeTe (0.037 × 10${}^{-10}$ C/m). The coexistence of ferromagnetism and piezoelectricity in the Cr${}_{2}$I${}_{3}$F${}_{3}$ monolayer, as a multi-functional 2D material, would greatly expand its application field in future nano-technology. Specifically, the Cr${}_{2}$I${}_{3}$F${}_{3}$ monolayer provides a promising way for the application of nanoscale spintronics and piezoelectric devices, such as catalysis, spintronics, energy harvesting, and pressure sensors.

## Figures and Tables

**Table 1:** Lattice parameter *a*, bond length *d${}_{1}$* and *d${}_{2}$*, and bond angle $\theta_{1}$ and $\theta_{2}$ of the monolayer CrI${}_{3}$, Cr${}_{2}$I${}_{3}$F${}_{3}$, and CrF${}_{3}$.

Monolayer	*a* (Å)	$d_{1}$ (Å)	$d_{2}$ (Å)	$\theta_{1}$ (${}^{\circ }$)	$\theta_{2}$ (${}^{\circ }$)
CrI${}_{3}$	7.002	2.736	2.736	95.25	95.25
Cr${}_{2}$I${}_{3}$F${}_{3}$	6.246	2.057	2.695	122.48	84.00
CrF${}_{3}$	5.181	1.941	1.941	100.75	100.75

**Table 2:** Piezoelectric coefficients (*e*${}_{ij}$, *d*${}_{ij}$) and elastic stiffness constants (*C*${}_{ij}$) of the Janus Cr${}_{2}$I${}_{3}$F${}_{3}$ monolayer. The corresponding coefficients of other Janus monolayers reported previously are listed for comparison as well. The units of *e*${}_{ij}$, *d*${}_{ij}$, and *C*${}_{ij}$ for these 2D materials are 10${}^{-10}$ C/m, pm/V, and N/m, respectively.

Pattern	*e* ${}_{31}$	*d* ${}_{31}$	*e* ${}_{11}$	*d* ${}_{11}$	*C* ${}_{11}$	*C* ${}_{12}$
Cr${}_{2}$I${}_{3}$F${}_{3}$	0.301	0.475	1.870	5.485	48.762	14.668
CrI${}_{3}$	∼	∼	∼	∼	26.378	6.516
MoS${}_{2}$ ^1^	∼	∼	3.64	3.73	130	32
MoSSe ^2,3^	0.032	0.020	3.74	3.76	126.78	20.61
In${}_{2}$SSe ^1^	0.13	0.18	3.24	8.47	55	17
MoSTe ^1^	0.038	0.028	4.53	5.036	112.7	22.7
MoSeTe ^2,3^	0.037	0.030	4.35	5.30	96.57	20.67
WSeTe ^2,3^	0.010	0.008	3.34	3.52	111.03	17.31
WSSe ^2,3^	0.018	0.011	2.57	2.26	141.27	22.41
WSTe ^2,3^	0.010	0.007	3.48	3.33	133.62	26.25

^1^ Ref. [43], ^2^ Ref. [15], ^3^ Ref. [44].

**Table 3:** The MAE (in unit of meV), the nearest neighbor (NN) exchange interaction *J*${}_{1}$, the next nearest neighbor (NNN) interaction *J*${}_{2}$, the third nearest neighbour (3NN) interaction *J*${}_{3}$ (in unit of meV), and the Curie temperature *T*${}_{C}$ (in unit of K) of the Cr${}_{2}$I${}_{3}$F${}_{3}$, CrF${}_{3}$, and CrI${}_{3}$ monolayer.

Pattern	MAE	*J* ${}_{1}$	*J* ${}_{2}$	*J* ${}_{3}$	*T* ${}_{C}$
CrF${}_{3}$	−0.130	1.750	0.066	−0.009	20.8
CrI${}_{3}$	−0.737	2.896	0.639	−0.154	56
Cr${}_{2}$I${}_{3}$F${}_{3}$	3.064	0.549	0.574	0.150	47

## Data Availability

Not applicable.

## References

[1] Jiang X., Liu Q.X., Xing J.P., Liu N.S., Guo Y., Liu Z.F., Zhao J.J. (2021). Recent progress on 2D magnets: Fundamental mechanism, structural design and modification. Appl. Phys. Rev..

[2] Song Q., Occhialini C.A., Ergecen E., Ilyas B., Amoroso D., Barone P., Kapeghian J., Watanabe K., Taniguchi T., Botana A.S. (2022). Evidence for a single-layer van der Waals multiferroic. Nature.

[3] Cui C., Xue F., Hu W.J., Li L.J. (2018). Two-dimensional materials with piezoelectric and ferroelectric functionalities. NPJ 2D Mater. Appl..

[4] Huang B., Clark G., Navarro-Moratalla E., Klein D.R., Cheng R., Seyler K.L., Zhong D., Schmidgall E., McGuire M.A., Cobden D.H. (2017). Layer-dependent ferromagnetism in a van der Waals crystal down to the monolayer limit. Nature.

[5] Sainbileg B., Batsaikhan E., Hayashi M. (2020). Impact of oxygen defects on a ferromagnetic CrI_3_ monolayer. RSC Adv..

[6] Tomar S., Ghosh B., Mardanya S., Rastogi P., Bhadoria B.S., Chauhan Y.S., Agarwal A., Bhowmick S. (2019). Intrinsic magnetism in monolayer transition metal trihalides: A comparative study. J. Magn. Magn. Mater..

[7] Zhang W.B., Qu Q., Zhua P., Lam C.H. (2015). Robust intrinsic ferromagnetism and half semiconductivity in stable two-dimensional single-layer chromium trihalides. J. Mater. Chem. C.

[8] Zhao Y.G., Lin L.F., Zhou Q.H., Li Y.H., Yuan S.J., Chen Q., Dong S., Wang J.L. (2018). Surface Vacancy-Induced Switchable Electric Polarization and Enhanced Ferromagnetism in Monolayer Metal Trihalides. Nano Lett..

[9] Zhang X.L., Yang Z.X., Chen Y. (2017). Novel two-dimensional ferroelectric PbTe under tension: A first-principles prediction. J. Appl. Phys..

[10] Hu T., Wu H.P., Zeng H.B., Deng K.M., Kan E. (2016). New Ferroelectric Phase in Atomic-Thick Phosphorene Nanoribbons: Existence of in-Plane Electric Polarization. Nano Lett..

[11] Wu M.H., Dong S., Yao K.L., Liu J.M., Zeng X.C. (2016). Ferroelectricity in Covalently functionalized Two-dimensional Materials: Integration of High-mobility Semiconductors and Nonvolatile Memory. Nano Lett..

[12] Huang C.X., Du Y.P., Wu H.P., Xiang H.J., Deng K.M., Kan E.J. (2018). Prediction of Intrinsic Ferromagnetic Ferroelectricity in a Transition-Metal Halide Monolayer. Phys. Rev. Lett..

[13] Li R.P., Cheng Y.C., Huang W. (2018). Recent Progress of Janus 2D Transition Metal Chalcogenides: From Theory to Experiments. Small.

[14] Wu Y., Yang C.H., Zhang H.N., Zhu L.H., Wang X.Y., Li Y.Q., Zhu S.Y., Wang X.C. (2022). The flexible Janus X_2_PAs (X = Si, Ge and Sn) monolayers with in-plane and out-of-plane piezoelectricity. Appl. Surf. Sci..

[15] Dong L., Lou J., Shenoy V.B. (2017). Large In-Plane and Vertical Piezoelectricity in Janus Transition Metal Dichalchogenides. ACS Nano.

[16] Zhang J., Jia S., Kholmanov I., Dong L., Er D.Q., Chen W.B., Guo H., Jin Z.H., Shenoy V.B., Shi L. (2017). Janus Monolayer Transition-Metal Dichalcogenides. ACS Nano.

[17] Maggay I.V.B., Yeh K.Y., Lei B.F., Brik M.G., Piasecki M., Liu W.R. (2018). Luminescence properties of Eu2+ -activated NaCaBeSi_2_O_6_F for white light-emitting diode applications. Mater. Res. Bull..

[18] Sherrell P.C., Fronzi M., Shepelin N.A., Corletto A., Winkler D.A., Ford M., Shapter J.G., Ellis A.V. (2022). A bright future for engineering piezoelectric 2D crystals. Chem. Soc. Rev..

[19] Song G., Zhang C.F., Zhang Z.Z., Li G.N., Li Z.W., Du J., Zhang B.W., Huang X.K., Gao B.L. (2022). Coexistence of intrinsic room-temperature ferromagnetism and piezoelectricity in monolayer BiCrX_3_ (X = S, Se, and Te). Phys. Chem. Chem. Phys..

[20] Tu S.C., Guo Y.X., Zhang Y.H., Hu C., Zhang T.R., Ma T.O.Y., Huang H.W. (2020). Piezocatalysis and Piezo-Photocatalysis: Catalysts Classification and Modification Strategy, Reaction Mechanism, and Practical Application. Adv. Funct. Mater..

[21] Wang R., Su Y., Yang G.H., Zhang J.F., Zhang S.B. (2020). Bipolar Doping by Intrinsic Defects and Magnetic Phase Instability in Monolayer CrI_3_. Chem. Mater..

[22] Zhang L., Yang Z.J.F., Gong T., Pan R.K., Wang H.D., Guo Z.A., Zhang H., Fu X. (2020). Recent advances in emerging Janus two-dimensional materials: From fundamental physics to device applications. J. Mater. Chem. A.

[23] Kresse G., Furthmuller J. (1996). Efficient iterative schemes for ab initio total-energy calculations using a plane-wave basis set. Phys. Rev. B Condens. Matter..

[24] Blochl P.E. (1994). Projector augmented-wave method. Phys. Rev. B Condens. Matter..

[25] Perdew J.P., Burke K., Ernzerhof M. (1996). Generalized Gradient Approximation Made Simple. Phys. Rev. Lett..

[26] Krukau A.V., Vydrov O.A., Izmaylov A.F., Scuseria G.E. (2006). Influence of the Exchange Screening Parameter on the Performance of Screened Hybrid Functionals. J. Chem. Phys..

[27] Marsman M., Paier J., Stroppa A., Kresse G. (2008). Hybrid Functionals Applied to Extended Systems. J. Phys. Condens. Matter.

[28] Wang V., Xu N., Liu J.C., Tang G., Geng W.T. (2021). VASPKIT: A user-friendly interface facilitating high-throughput computing and analysis using VASP code. Comput. Phys. Commun..

[29] Mostofi A.A., Yates J.R., Lee Y.S., Souza I., Vanderbilt D., Marzari N. (2008). wannier90: A tool for obtaining maximally-localised Wannier functions. Comput. Phys. Commun..

[30] Parlinski K., Li Z.Q., Kawazoe Y. (1997). First-Principles Determination of the Soft Mode in Cubic *ZrO*_2_. Phys. Rev. Lett..

[31] Togo A., Tanaka I. (2015). First Principles Phonon Calculations in Materials Science. Scr. Mater..

[32] Liu L., Chen S.S., Lin Z.Z., Zhang X. (2020). A Symmetry-Breaking Phase in Two-Dimensional FeTe2 with Ferromagnetism above Room Temperature. J. Phys. Chem. Lett..

[33] Liu L., Ren X., Xie J.H., Cheng B., Liu W.K., An T.Y., Qin H.W., Hu J.F. (2019). Magnetic switches via electric field in BN nanoribbons. Appl. Surf. Sci..

[34] Vanderbilt D., King-Smith R.D. (1993). Electric polarization as a bulk quantity and its relation to surface charge. Phys. Rev. B Condens. Matter..

[35] Korir K.K., Cicero G., Catellani A. (2013). Piezoelectric properties of zinc oxide nanowires: An ab initio study. Nanotechnology.

[36] Cicero G., Ferretti A., Catellani A. (2009). Surface-induced polarity inversion in ZnO nanowires. Phys. Rev. B.

[37] Qin W., Xu B., Liao S., Liu G., Sun B., Wu M. (2020). Flat-band splitting induced tunable magnetism in defective CrI_3_ monolayer. Solid State Commun..

[38] Zhang J.Y., Zhao B., Zhou T., Xue Y., Ma C.L., Yang Z.Q. (2018). Strong magnetization and Chern insulators in compressed graphene/CrI_3_ van derWaals heterostructures. Phys. Rev. B.

[39] Zheng F.W., Zhao J.Z., Liu Z., Li M.L., Zhou M., Zhang S.B., Zhang P. (2018). Tunable spin states in the two-dimensional magnet CrI_3_. Nanoscale.

[40] Webster L., Yan J.A. (2018). Strain-tunable magnetic anisotropy in monolayer CrCl_3_, CrBr_3_, and CrI_3_. Phys. Rev. B.

[41] Pei Q., Zhou B.Z., Mi W.B., Cheng Y.C. (2019). Triferroic Material and Electrical Control of Valley Degree of Freedom. ACS Appl. Mater. Interfaces.

[42] Wang V., Geng W.T. (2017). Lattice Defects and the Mechanical Anisotropy of Borophene. J. Phys. Chem. C.

[43] Chen Y., Liu J.Y., Yu J.B., Guo Y.G., Sun Q. (2019). Symmetry-breaking induced large piezoelectricity in Janus tellurene materials. Phys. Chem. Chem. Phys..

[44] Thanh V.V., Van N.D., Truong D.V., Saito R., Hung N.T. (2020). First-principles study of mechanical, electronic and optical properties of Janus structure in transition metal dichalcogenides. Appl. Surf. Sci..

[45] Pham T.H., Ullah H., Shafique A., Kim H.J., Shin Y.H. (2021). Enhanced out-of-plane electromechanical response of Janus ZrSeO. Phys. Chem. Chem. Phys..

[46] McGuire M.A., Dixit H., Cooper V.R., Sales B.C. (2015). Coupling of Crystal Structure and Magnetism in the Layered, Ferromagnetic Insulator CrI_3_. Chem. Mater..

[47] Ares P., Cea T., Holwill M., Wang Y.B., Roldan R., Guinea F., Andreeva D.V., Fumagalli L., Novoselov K.S., Woods C.R. (2020). Piezoelectricity in Monolayer Hexagonal Boron Nitride. Adv. Mater..

[48] Zhu H.Y., Wang Y., Xiao J., Liu M., Xiong S.M., Wong Z.J., Ye Z.L., Ye Y., Yin X.B., Zhang X. (2015). Observation of piezoelectricity in free-standing monolayer MoS_2_. Nat. Nanotechnol..

[49] Bottom V.E. (1970). Measurement of piezoelectric coefficient of quartz using fabry-perot dilatometer. J. Appl. Phys..

[50] Vurgaftman I., Meyer J.R. (2003). Band parameters for nitrogen-containing semiconductors. J. Appl. Phys..

[51] Zhang Y., Ye H., Yu Z., Liu Y., Li Y. (2019). First-principles study of square phase MX_2_ and Janus MXY (M = Mo, W; X, Y = S, Se, Te) transition metal dichalcogenide monolayers under biaxial strain. Phys. E Low-Dimens. Syst. Nanostruct..

[52] Zhang Y., Liu C.H., Liu J.B., Xiong J., Liu J.Y., Zhang K., Liu Y.D., Peng M.Z., Yu A.F., Zhang A.H. (2016). Lattice Strain Induced Remarkable Enhancement in Piezoelectric Performance of ZnO-Based Flexible Nanogenerators. ACS Appl. Mater. Interfaces.

[53] Liu H., Chen J., Fan L.L., Ren Y., Pan Z., Lalitha K.V., Rodel J.G., Xing X.R. (2017). Critical Role of Monoclinic Polarization Rotation in High-Performance Perovskite Piezoelectric Materials. Phys. Rev. Lett..

[54] Yang J., Wang A., Zhang S., Liu J., Zhong Z., Chen L. (2018). Coexistence of piezoelectricity and magnetism in two-dimensional vanadium dichalcogenides. Phys. Chem. Chem. Phys..

[55] Guo S.D., Zhu Y.T., Mu W.Q., Ren W.C. (2020). Intrinsic piezoelectricity in monolayer MSi_2_N_4_ (M = Mo, W, Cr, Ti, Zr and Hf). EPL (Europhys. Lett.).

[56] Jena N., Rawat A., Ahammed R., Mohanta M.K., De Sarkar A. (2018). Emergence of high piezoelectricity along with robust electron mobility in Janus structures in semiconducting Group IVB dichalcogenide monolayers. J. Mater. Chem. A.

